# Why do patients follow physicians’ advice? The influence of patients’ regulatory focus on adherence: an empirical study in China

**DOI:** 10.1186/s12913-019-4127-9

**Published:** 2019-05-10

**Authors:** Runtong Zhang, Xinyi Lu, Wen Wu, Xiaopu Shang

**Affiliations:** School of Economics and Management, Beijing Jiaotong University, Shangyuancun 3, Haidian District, Beijing, 100044 China

**Keywords:** Regulatory focus, Patient adherence, Physician-patient relationship, Health information seeking, Structural equation modelling

## Abstract

**Background:**

In general, medical regimens and treatments are more likely to be effective if patients follow their physicians’ advice. However, limited studies have focused on the relationship between regulatory focus and patient adherence. This study explores the antecedents of patient adherence employing regulatory focus theory.

**Methods:**

This study established a research model consisting of two independent variables, two mediators, one dependent variable, two moderators, three control variables, and six hypotheses. An online survey involving 336 valid responses was conducted to collect data in China. We used structural equation modelling and confirmatory factor analysis to test the hypotheses and to develop the research model.

**Results:**

The reliability and validity of the measures were accepted. In terms of control variables, age had a positive effect on conservative treatment-related health information seeking behaviour, and patients with different resident statuses held different attitudes towards seeking conservative treatment-related health information. However, educational level did not have any effect on the variables of the research model. The hypothesis testing results corroborate that promotion focus had a positive impact on patients’ emerging treatment-related health information seeking behaviour; prevention focus had a positive impact on patients’ conservative treatment-related health information seeking behaviour, which had a positive impact on patient adherence. In addition, media campaigns had a positive impact on the relationship between promotion focus and emerging treatment-related health information seeking behaviour, and website reputation had a positive impact on the relationship between prevention focus and conservative treatment-related health information seeking behaviour.

**Conclusions:**

Individuals can be encouraged to seek health information and share health-related knowledge through mass media, such as the Internet, when the quality of information, especially information from online sources, is guaranteed. In addition, physicians need to improve their professionalism and expand their knowledge of conservative healthcare. As a further application of our work, an Internet information recommendation system can be designed to recommend different types of health information for users according to their regulatory focus.

**Electronic supplementary material:**

The online version of this article (10.1186/s12913-019-4127-9) contains supplementary material, which is available to authorized users.

## Background

The physician-patient relationship has a significant effect on diagnoses and treatments [1]. In general, medical regimens and treatments are more likely to be effective if patients follow physicians’ advice [2], and the quality of the physician-patient relationship plays an important role in improving patient adherence [3]. Adherence (or compliance) is defined as “the extent to which a person’s behaviour (in terms of taking medications, following diets, or executing lifestyle changes) coincides with medical or health advice” [4]. Healthcare providers prefer to use “adherence” because “compliance” tends to describe the degree to which patients passively follow physicians’ orders and treatments that are formulated by physicians themselves without consulting with patients [5]. Patients with considerably high adherence are likely to become healthier than those with poor adherence [6], and nonadherence may result in several serious financial, social, familial, individual, and psychological problems [7], such as low income due to an inability to work, high medical costs, and even health deterioration [2]. With the increasing proportion of chronic diseases [8, 9], patient self-management has become critical in treatments [10]. Thus, patient adherence is particularly important for health and rehabilitation.

Previous studies have suggested that patient adherence should be regarded as a dynamic parameter. In this view, nonadherence to physicians’ instructions and treatments may be involuntary. That is, patients may be distracted and forgetful because of certain factors in the treatment process [11]. While involuntary nonadherence may be related to individual characteristics, the rational decision-making process is reflected by voluntary nonadherence.

Some studies have focused on patient adherence and its corresponding influencing factors and consequences. These studies discuss four main aspects: (1) the relationships between patient adherence and various diseases [12–14]; (2) the improvement and optimization of patient adherence [15, 16]; (3) the factors influencing patient adherence [17–19]; and (4) medicine adherence [20, 21]. However, related previous studies that explored the relationship between regulatory focus and patient adherence are limited. Thus, the present study investigates the antecedents of patient adherence by employing regulatory focus theory.

Before Crowe and Higgins [22] proposed regulatory focus theory, the pleasure principle had been used as the basic motivational assumption across all fields of psychology to understand individual motivation. The pleasure principle was replaced by regulatory focus theory, which is based on self-discrepancy theory [23] and involves two types of regulatory focus, promotion focus and prevention focus, which are characterized by approach and avoidance motivations, respectively. Promotion focus is concerned with advancement, growth, and accomplishment, and it has a strategic inclination towards achieving progress and approaching the desired end-state. Approach motivation aims to achieve the ideals and aspirations of individuals. It focuses on personal development and self-realization and expects positive results. By contrast, prevention focus is concerned with security, safety, and responsibility, and it has a strategic inclination towards being prudent and precautionary to avoid mismatches with the desired end-state [22]. Avoidance motivation aims to avoid failures and mistakes, to fulfil the responsibilities and obligations of individuals, and to meet the expectations of others without negative results [24].

Apart from physicians, patients also obtain health information from friends, news, books, the Internet and other sources [2]. The health information acquired by patients from daily life may exert both positive and negative effects on the physician-patient relationship. Positive effects are generated when the obtained health information strengthens the confidence of patients in their physicians, encourages patients to make suitable choices and decisions, improves their understanding of their health status, and enhances the physician-patient relationship [25, 26]. However, some patients seek health information because they are dissatisfied or discontented with their physicians. In addition, some physicians believe that their patients cannot assess the reliability of health information, and a minority of physicians may object to their patients seeking other information because they feel challenged, resulting in physician hostility, low healthcare quality, anxiety, and frustration among patients [26, 27]. A previous survey [27] indicated that physicians generally admitted that health information can significantly help improve patients’ health; however, 40% of physicians worried that such health information might adversely affect the physician-patient relationship.

Information behaviour refers to human behaviour that is related to information resources and information channels, including active or passive information seeking behaviour, information utilization behaviour, face-to-face communication behaviour, and passive information receiving behaviour. Information seeking behaviour includes, but is not limited to, two main types of information behaviours: information retrieval and information browsing. Wilson [28] defined information seeking behaviour as a purposeful activity to seek information to satisfy a certain target requirement. Its objective is to meet the information needs of patients and to reduce the information asymmetry between physicians and patients [29]. Patients obtain health information through information seeking behaviour and feel a significant increase in their level of knowledge by reading information [2]. However, Laugesen et al. [2] argued that patients sometimes realize that they access only basic information that physicians have already known when they interact with physicians; hence, the perceived information asymmetry still exists. Therefore, a change in patients’ perceived information asymmetry is related to the nature of the health information obtained through information seeking behaviour.

This study explores how regulatory focus affects patient adherence through the mediation of information seeking behaviour. Considering that information seeking behaviour is ultimately influenced by different individual characteristics, we assume that patients with different regulatory focuses exhibit different health information seeking behaviours. This difference may produce various information gaps between physicians and patients, thereby affecting patient adherence [2].

## Research model and hypotheses

Regulatory focus theory suggests that individuals with different regulatory focuses constantly perform differently. Specifically, individuals in a promotion focus state are willing to obtain emerging treatment-related information, whereas individuals in a prevention focus state are “conservative” and often make repetitive choices or follow routines to avoid risks. The uncertainty of technical effectiveness makes individuals with a prevention focus hesitant to adopt emerging technology, prompting them to follow previous behaviour rather than innovate [30]. Therefore, we derive the following hypotheses:

- **H1**: Promotion focus has a positive impact on patients’ emerging treatment-related health information seeking behaviour.
- **H2**: Prevention focus has a positive impact on the patients’ conservative treatment-related health information seeking behaviour.

Patients with a promotion focus are more likely to seek emerging treatment-related health information; their physicians may lack such information because of its “emerging” nature. Through information seeking behaviour, patients may gain much health information. Thus, their perceived information asymmetry may be reduced. By contrast, patients with a prevention focus often obtain conservative treatment-related health information that is already known by physicians. This situation further convinces patients that physicians grasp more information than they do. As a result, patients believe that their physicians have professional knowledge and eventually perceive the increase in information asymmetry. The greater the amount of effective information that patients acquire is, the greater the extent to which they trust in their physicians during the interaction. Laugesen et al. [2] proposed that the greater the information asymmetry, the better the patient adherence. This situation leads to the following hypotheses:

- **H3**: The emerging treatment-related health information seeking behaviour of patients with a promotion focus has a negative impact on their adherence.
- **H4**: The conservative treatment-related health information seeking behaviour of patients with a prevention focus has a positive impact on their adherence.

Mass media, which include television, newspapers, and the Internet, have become an important source of information related to healthy lifestyles. Mass media exert a significant effect on spreading and seeking health information [31], and they play an important role in early diagnoses and disease management [32]. In consideration of the decrease in the credibility of media campaigns [33, 34], patients with a prevention focus are less likely to use mass media than those with a promotion focus. Emerging treatment-related health information is mainly disseminated to the public through mass media. Thus, media campaigns may encourage patients with a promotion focus to seek emerging treatment-related health information. The above discussion leads us to propose the following hypothesis:

- **H5**: If the level of media campaigns increases, then the relationship between promotion focus and emerging treatment-related health information seeking behaviour will be strengthened.

As a main source of health information, the Internet provides a quick and convenient way for individuals to seek health information. Patients with a prevention focus tend to obtain conservative treatment-related health information after much deliberation, for example, by comparing the same information from different websites. Website reputation is produced and diffused online. From the perspective of users, websites with a high reputation are more likely to have high credibility, and reputation is critical in constructing trust between websites and users [35]. Therefore, patients with a prevention focus are more likely to seek conservative treatment-related health information on websites with a high level of reputation. Consequently, we suggest the following hypothesis:

- **H6**: If the level of website reputation increases, then the relationship between prevention focus and conservative treatment-related health information seeking behaviour will be strengthened.

**Fig. 1:**
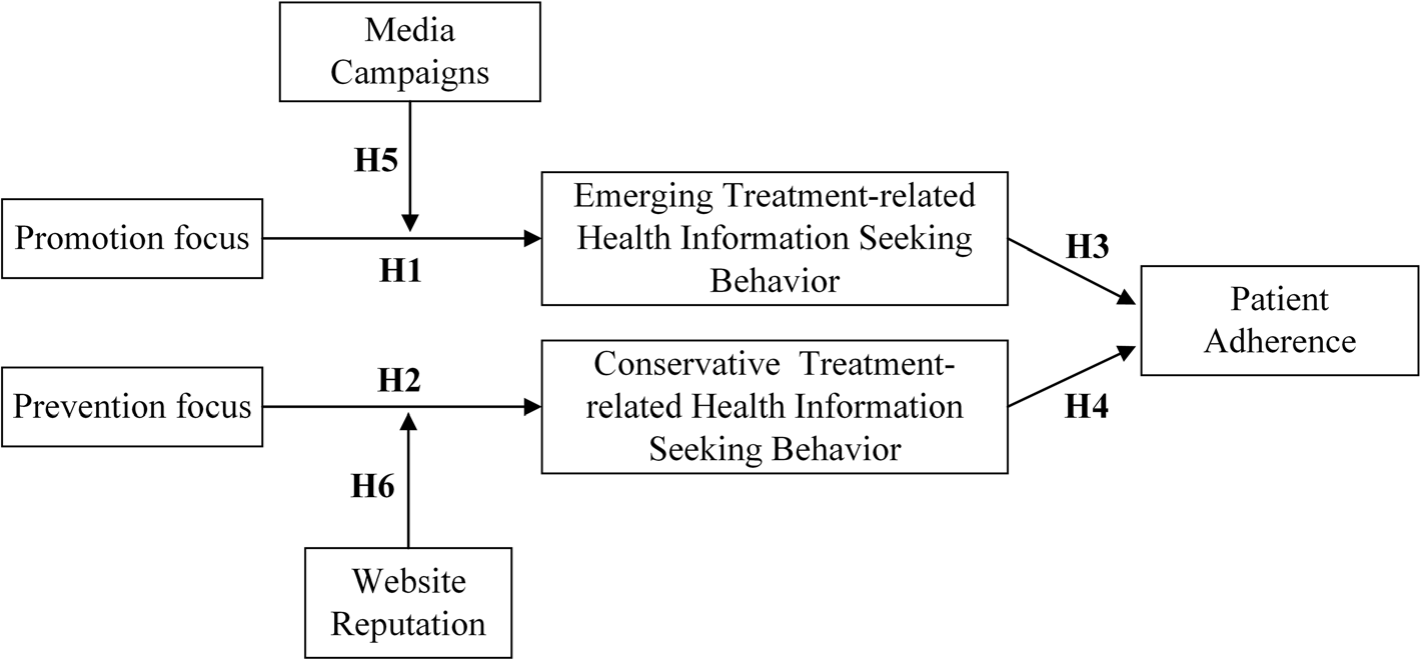
Research model

Our research model (see Fig. 1) is established by summarizing the above hypotheses, with promotion focus and prevention focus as independent variables, emerging treatment-related health information seeking behaviour and conservative treatment-related health information seeking behaviour as mediators, patient adherence as the dependent variable, and media campaigns and website reputation as moderators.

## Methods

### Participants

The participants in this study were Chinese individuals who had experiences seeking health information and going to hospitals within the previous month so that they could recall (1) their experiences in seeking health information related to their specific health conditions and (2) their experiences and feelings with regard to their treatments.

### Measurement instrument

All variables in this research model (see Fig. 1) were measured by multiple-item scales widely used in previous studies to ensure significant reliabilities and validities. A 7-point Likert-type response format ranging from “strongly disagree” to “strongly agree” was used to measure all items. Additional file 1 provides a list of questionnaire items. Promotion focus and prevention focus were measured using two nine-item scales adopted from Lockwood et al. [36]. Emerging treatment-related and conservative treatment-related health information seeking behaviours were measured using a five-item scale adapted based on items from Lemire et al. [37]. In this study, patient adherence was measured using a five-item scale adapted by Laugesen et al. [2] based on items from Hausman [38]. As a moderator, media campaigns were used to evaluate the attitudes of individuals towards health information obtained through mass media, and they were measured using a 15-item scale adopted from Lignowska et al. [39]. A four-item scale adopted from Li [40] was used to measure website reputation.

### Procedure

Prior to the formal investigation, the questionnaire was translated from English into Chinese because our subjects were Chinese. First, we translated the scales into Chinese and modified some expressions for cross-cultural adaptation. Then, we invited 10 individuals from different fields of work with different ages, genders and educational levels to read the translated scales and provide recommendations to further modify them. This process ensured the readability and comprehensibility of the scales. Finally, the Chinese scales were translated back into English by an English-speaking professional to ensure consistency between the new English translation versions and the original versions.

An online questionnaire survey was conducted in June 2017. The questionnaire was completed anonymously. A statement on the front page of the questionnaire clarified the protection of the participants’ privacy, and the participants’ informed consent was secured. Each piece of equipment was allowed to fill in the questionnaire only once to avoid duplication. In the weeks preceding the survey, this questionnaire was pre-tested on 112 subjects to ensure that the scales were clear, concise, and easy to read and to determine the approximate time to complete the questionnaire. With the help of a medical association in China, we sent questionnaires to 486 participants and obtained 375 responses, 336 of which were valid. Therefore, the response rate was 77.16%, and the valid response rate was 89.6%. Table 1 shows the demographics of the valid responses.

**Table 1:** Demographics of the sample

	Number	Percentage
(1) Age		
< 20	22	6.55%
21–29	83	24.70%
30–39	107	31.84%
40–49	59	17.56%
50–59	47	13.99%
60 and above	18	5.36%
(2) Gender		
Male	156	46.43%
Female	180	53.57%
(3) Resident status		
Urban	184	54.76%
Rural	152	45.24%
(4) Education		
Junior middle school	31	9.22%
High school	96	28.57%
Junior college	68	20.24%
Bachelor’s degree	127	37.80%
Master’s degree	9	2.68%
Doctoral degree	5	1.49%

A previous study indicated that a high proportion of individuals who are likely to search the Internet for health information are young and highly educated females [2]. In our study, the Internet was not the only platform that could be used to seek health information; website reputation was also a moderator, and this survey was conducted through the Internet. Thus, the participants in this study met the requirements.

## Results

### Data analysis

This study drew lessons from some research methods in previous studies [3, 41]. Structural equation modelling (SEM) is a widely accepted paradigm [42] that can be used to test research models, including all variables, and to analyse the causal relations between model parameters [43]. Thus, it is suitable for use to analyse a research model containing mediators, moderators and complex relationships. Statistical analysis was performed using SPSS 22.0. The reliability of the measures was assessed by Cronbach’s α, which should be at least 0.70 [44]. Table 2 shows the Cronbach’s α of each construct, which means acceptable reliability. The KMO (Kaiser-Meyer-Olkin) value (weak: 0.50; medium: 0.60; good: 0.70; very good: 0.80; perfect: 0.90) [45–48] was equal to 0.85 (*p* < 0.001, significant) and above the cut-off value of 0.80. Therefore, the data collected through our questionnaires were suitable for factor analysis.

**Table 2:** Cronbach’s α of the constructs

Construct^a^	Cronbach’s α
PROF	0.80
PREF	0.76
ETHISB	0.84
CTHISB	0.86
MC	0.83
WR	0.91
PA	0.87
Total	0.85

^a^*PROF* promotion focus, *PREF* prevention focus, *ETHISB* emerging treatment-related health information seeking behaviour, *CTHISB* conservative treatment-related health information seeking behaviour, *MC* media campaigns, *WR* website reputation, *PA* patient adherence

We used confirmatory factor analysis (CFA) and SEM to analyse the data and to test the hypotheses. Based on the CFA results from SPSS AMOS 22.0, we deleted three promotion focus items (PROF3, PROF4 and PROF6), four prevention focus items (PREF2, PREF4, PREF5 and PREF7) and two media campaign items (MC7 and MC15) with low loadings (< 0.50) or high cross-loadings (> 0.40). As shown in Table 3, the fit indices indicated a good fit and showed a consistency between the hypothesized model and the questionnaire data.

**Table 3:** Cronbach’s α of the constructs

Fit indices	Values	Threshold for a good fit [49]
Root mean square error of approximation (RMSEA) [50]	0.03	< 0.05
Goodness of fit index (GFI) [51]	0.90	≥0.90
Comparative fit index (CFI) [52]	0.96	≥0.90

Table 4 provides the mean, standard deviation (SD), composite reliability (CR), and average variance extracted (AVE), and Table 5 presents the correlations between each of the two factors. The AVE and CR were used to evaluate convergent validity. For almost each measure, the AVE was above the cut-off value of 0.50 and the CR was above the cut-off value of 0.70, indicating that the measures had an acceptable convergent validity. Discriminant validity was acceptable because the square root of the AVE of almost every construct exceeded the correlations between the other constructs in the model and itself (except for the correlation between promotion focus and prevention focus) [53, 54].

**Table 4:** Composite reliability and average variance extracted

Construct^a^	Mean	SD	CR	AVE	Sqrt AVE
PROF	5.12	0.80	0.84	0.47	0.68
PREF	4.97	0.82	0.83	0.50	0.70
ETHISB	4.21	0.92	0.87	0.57	0.75
CTHISB	4.68	0.83	0.86	0.55	0.74
MC	3.63	0.69	0.91	0.53	0.73
WR	5.09	1.12	0.89	0.68	0.82
PA	5.25	0.85	0.88	0.60	0.78

^a^*PROF* promotion focus, *PREF* prevention focus, *ETHISB* emerging treatment-related health information seeking behaviour, *CTHISB* conservative treatment-related health information seeking behaviour, *MC* media campaigns, *WR* website reputation, *PA* patient adherence

**Table 5:** Correlations between two constructs

Construct^a^	PROF	PREF	ETHISB	CTHISB	MC	WR	PA
PROF	1.00						
PREF	0.85	1.00					
ETHISB	0.41	0.29	1.00				
CTHISB	0.31	0.41	0.30	1.00			
MC	0.03	0.13	0.22	0.17	1.00		
WR	0.35	0.32	0.31	0.34	−0.18	1.00	
PA	0.33	0.25	0.15	0.34	−0.23	0.38	1.00

^a^*PROF* promotion focus, *PREF* prevention focus, *ETHISB* emerging treatment-related health information seeking behaviour, *CTHISB* conservative treatment-related health information seeking behaviour, *MC* media campaigns, *WR* website reputation, *PA* patient adherence

### Hypothesis testing

**Fig. 2:**
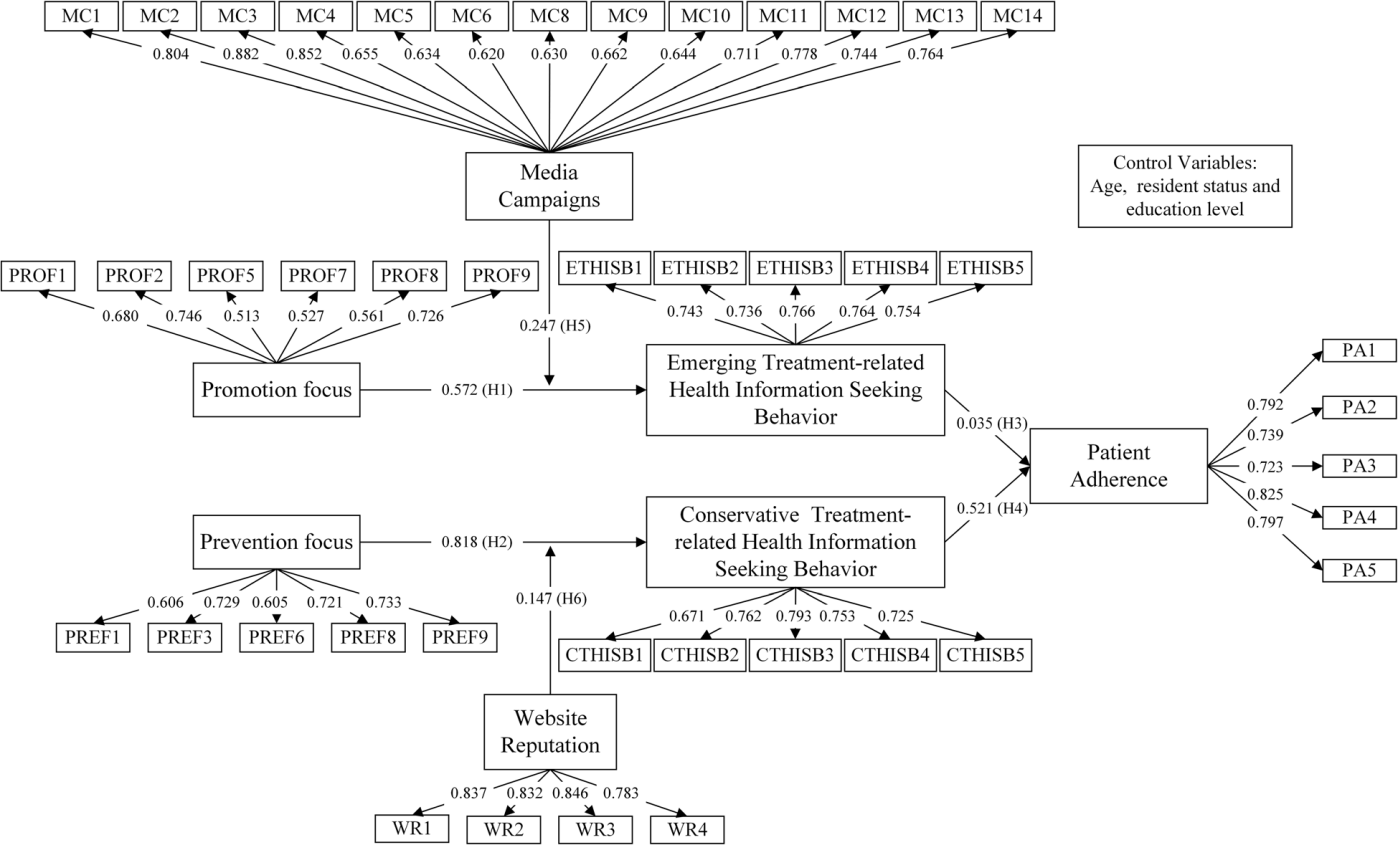
Research model with path coefficients

First, we analysed the effects of demographic factors (age, gender, resident status, and educational level) on the research model using a t-test, analysis of variance (ANOVA) and analysis of covariance (ANOCOVA). We found that age, resident status, and educational level exerted significant effects on these relationships. Therefore, we added these variables into the research model as control variables. The results revealed that age exerted a positive effect on conservative treatment-related health information seeking behaviour and that patients with different resident statuses held different attitudes towards seeking conservative treatment-related health information. Educational level did not have any effect on the variables of the research model. Figure 2 and Table 6 show the magnitude and significance of the path coefficients. It is found that five hypotheses (**H1**, **H2**, **H4**, **H5**, and **H6**) were supported. The impact directions of all supported hypotheses were consistent with the path coefficients. **H3** was not supported, and the path coefficient between emerging treatment-related health information seeking behaviour and patient adherence was not significant because it was near zero. To further test the mediating effects, we used the bootstrapping method (*n* = 1000, 95% CI) with SPSS AMOS 22.0. We found that conservative treatment-related health information seeking behaviour played a total mediating role between prevention focus and patient adherence [55].

**Table 6:** Results of hypothesis testing

Hypotheses	Path coefficient	P
Promotion focus has a positive impact on patients’ emerging treatment-related health information seeking behaviour.	0.527	< 0.001
Prevention focus has a positive impact on the patients’ conservative treatment-related health information seeking behaviour.	0.818	< 0.001
The emerging treatment-related health information seeking behaviour of patients with a promotion focus has a negative impact on their adherence.	0.035	0.593
The conservative treatment-related health information seeking behaviour of patients with a prevention focus has a positive impact on their adherence.	0.521	< 0.001
If the level of media campaigns increases, then the relationship between promotion focus and emerging treatment-related health information seeking behaviour will be strengthened.	0.247	< 0.001
If the level of website reputation increases, then the relationship between prevention focus and conservative treatment-related health information seeking behaviour will be strengthened.	0.147	0.011

## Discussion

### Principal results

This study identified how regulatory focus influences patient adherence through the mediation of health information seeking behaviour and considered the moderating effects of media campaigns and website reputation on this relationship. The hypothesis testing results indicate that we can substantially ascertain (1) the relationship between regulatory focus and health information seeking behaviour, (2) the relationship between the conservative treatment-related health information seeking behaviour of patients with a prevention focus and patient adherence, and (3) the moderating effects of media campaigns and website reputation on the relationship between regulatory focus and health information seeking behaviour.

The development of the Internet allows people to freely post and review health-related topics online. Consistent with our hypothesis, regulatory focus strongly affects health information seeking behaviour. Specifically, individuals with a promotion focus are more likely to seek emerging treatment-related health information, whereas individuals with a prevention focus prefer conservative treatment-related health information because incautious people accept more risk while conservative people accept less risk [30]. Therefore, a system that recommends Internet health information for users according to their regulatory focus and risk preference may be designed. For example, users can answer some questions so that their regulatory focus and risk preference can be preliminarily judged. On the basis of their regulatory focus and some keywords input by users, such a system can provide appropriate health information for users based on some algorithms. Our findings also show the differences in health information seeking behaviours among different age groups. Thus, age may be an important factor that should be included in such a system.

Conservative treatment-related health information seeking behaviour is an important factor affecting patient adherence. Contradicting our hypothesis, the emerging treatment-related health information seeking behaviour of patients with a promotion focus did not have any significant effect on patient adherence. Additional analyses were performed to explore why **H3** was not supported. Considering that other factors may inhibit this relationship, we intended to analyse the direct effect of emerging treatment-related health information seeking behaviour on patient adherence without considering their other effects on patient adherence. The path from conservative treatment-related health information seeking behaviour to patient adherence was disregarded. Consequently, emerging treatment-related health information seeking behaviour significantly affected patient adherence. However, the impact was positive, but not negative as **H3** hypothesized. This finding may be attributed to the lack of accurate definitions for emerging treatment-related health information and conservative treatment-related health information in the questionnaire, which resulted in possible confusion between these two types of health information. In other words, conservative treatment-related health information may also be emerging, and vice versa, which possibly resulted in confusion. Considering this reason, we have not clarified the specific relationship between emerging treatment-related health information seeking behaviour and patient adherence. Nevertheless, we still believe that emerging treatment-related health information may affect patient adherence. For example, when a physician advises a patient to use a new technology, individuals with a prevention focus may be hesitant to follow the physician’s advice [30], whereas individuals with a promotion focus may be interested in the new technology. Therefore, patients will tend to seek further information about it and change their adherence.

Despite the confusion between conservative treatment-related and emerging treatment-related health information during the survey, we can ensure that conservative treatment-related health information seeking behaviour has a positive effect on patient adherence. In other words, conservative treatment-related health information is a preferred type of health information. Thus, we should pay more attention to this type of health information. On the one hand, in terms of the quality of conservative treatment-related health information, we should strengthen its supervision and take measures to improve it. Physicians, professionals, and investigators can be invited to review the quality of conservative treatment-related health information. On the other hand, physicians should be required to improve their professionalism [2] and expand their knowledge of conservative healthcare to widen the gap between the conservative treatment-related health information obtained by patients from the Internet and that acquired from physicians, thereby increasing patients’ perceived information asymmetry and thus improving patient adherence.

Additionally, we identified the significant moderating effects of (1) media campaigns on the relationship between promotion focus and emerging treatment-related health information seeking behaviour and (2) website reputation on the relationship between prevention focus and conservative treatment-related health information seeking behaviour. These results are consistent with our assumptions. The additional analyses indicate the direct effects of (1) media campaigns on emerging treatment-related health information seeking behaviour and (2) website reputation on conservative treatment-related health information seeking behaviour, which we did not hypothesize. The number of health information portals established by governments, medical institutions, and business corporations has significantly increased [56]. Moreover, obtaining information through mass media such as the Internet has become a lifestyle. Thus, individuals can be encouraged to seek health information and share health-related knowledge on the Internet as long as the quality of information is guaranteed.

### Limitations

The limitations of this study must be considered. First, we focused on patient adherence, which is only one aspect of the physician-patient relationship. Other factors concerning this relationship may also warrant investigation. Second, we did not identify the differences between the independent variables and the moderators. Specifically, media campaigns and website reputation were moderators in our research model, but they could also be considered independent variables in further analyses. Third, all concepts and relationships were measured only once. Thus, this study was conducted from a static perspective, which may be why the convergent validity of the correlation between promotion focus and prevention focus was unacceptable, even though we adopted the scale from previous valid works. Therefore, future studies can conduct the survey more than once. Fourth, all conclusions in this study are based on relationships between questionnaire responses in the absence of external validation. Finally, this study simplified a complex issue with many dimensions. For example, we abstracted the research background of patient adherence. Future studies can address the specific disease to accurately identify the impact of regulatory focus on patient adherence.

## Conclusion

Our findings suggest that conservative treatment-related health information is the most important element in our research model and that regulatory focus influences patient adherence through health information seeking behaviour. Specifically, regulatory focus has two aspects: promotion focus and prevention focus. Individuals with a promotion focus tend to seek emerging treatment-related health information, whereas individuals with a prevention focus tend to seek conservative treatment-related health information. The above findings suggest that conservative treatment-related health information may dominate the impact on patient adherence and that conservative treatment-related health information seeking behaviour is always influenced by prevention focus. Aside from the moderating effects, media campaigns and website reputation also directly affect health information seeking behaviour. Individuals are encouraged to seek health information and share health-related knowledge through mass media such as the Internet when the quality of information, especially information from online sources, is guaranteed. Moreover, traditional mass media and online sources should improve the quality of information to attract individuals to seek health information. Conservative treatment-related health information may be the most noteworthy.

## Additional file

Additional file 1: **Table S1.** Measurement Instrument. (DOCX 19 kb)

## Abbreviations

AVE: Average variance extracted
CFA: Confirmatory factor analysis
CFI: Comparative fit index
CR: Composite reliability
CTHISB: Conservative Treatment-related Health Information Seeking Behaviour
ETHISB: Emerging Treatment-related Health Information Seeking Behaviour
KMO: Kaiser-Meyer-Olkin
MC: Media Campaigns
NFI: Normed fit index
PA: Patient Adherence
PREF: Prevention focus
PROF: Promotion focus
RMSEA: Root mean square error of approximation
SD: Standard deviation
SEM: Structural equation modeling
SPSS: Statistic Package for Social Science
WR: Website Reputation
