# Supplementary material for: Why do patients follow physicians’ advice? The influence of patients’ regulatory focus on adherence: an empirical study in China
Source: BMC Health Serv Res. 2019 May 10;19:301. doi: 10.1186/s12913-019-4127-9 (PMC6511214; doi:10.1186/s12913-019-4127-9)
Supplement: Supplementary file 1 — Table S1. Measurement Instrument. (DOCX 19 kb) [file 12913_2019_4127_MOESM1_ESM.docx]

Additional file 1: **Table S1** Measurement Instrument

| **Construct** | **Items** |
| --- | --- |
| Promotion Focus | 1. I frequently imagine how I will achieve my hopes and aspirations.  2. I often think about the person I would ideally like to be in the future.  3. I typically focus on the success I hope to achieve in the future.  4. I often think about how I will achieve academic or work success.  5. My major goal right now is to achieve my academic or work ambitions.  6. I see myself as someone who is primarily striving to reach my “ideal self”-to fulfill my hopes, wishes, and aspirations.  7. In general, I am focused on achieving positive outcomes in my life.  8. I often imagine myself experiencing good things that I hope will happen to me.  9. Overall, I am more oriented toward achieving success than preventing failure. |
| Prevention Focus | 1. In general, I am focused on preventing negative events in my life.  2. I am anxious that I will fall short of my responsibilities and obligations.  3. I often think about the person I am afraid I might become in the future.  4. I often worry that I will fail to accomplish my academic or work goals.  5. I often imagine myself experiencing bad things that I fear might happen to me.  6. I frequently think about how I can prevent failures in my life.  7. I am more oriented toward preventing losses than I am toward achieving gains.  8. My major goal in school right now is to avoid becoming an academic or a work failure.  9. I see myself as someone who is primarily striving to become the self I “ought” to be—to fulfill my duties, responsibilities, and obligations. |
| Emerging Treatment-related Health Information Seeking Behaviour | 1. I frequently seek emerging health information to understand a health problem or an illness.  2. I frequently seek emerging health information to obtain different points of view from those offered by mainstream medicine.  3. I frequently seek emerging health information to prevent illness by adopting a healthy lifestyle.  4. I frequently seek emerging health information to find a specific solution to or treatment for a health problem.  5. I frequently seek emerging health information to help a friend or family member who is ill. |
| Conservative Treatment-related Health Information Seeking Behaviour | 1. I frequently seek conservative health information to understand a health problem or an illness.  2. I frequently seek conservative health information to obtain different points of view from those offered by mainstream medicine.  3. I frequently seek conservative health information to prevent illness by adopting a healthy lifestyle.  4. I frequently seek conservative health information to find a specific solution to or treatment for a health problem.  5. I frequently seek conservative health information to help a friend or family member who is ill. |
| Media Campaigns | 1. When I encounter health information in media campaigns, I feel aversion.  2. Health information in media campaigns annoys me.  3. I try to avoid health information disseminated in media campaigns.  4. When I encounter health information in media campaigns, I pay no attention to it.  5. When I encounter health information in media campaigns, I suspect that someone is trying to sell me some products.  6. Health popularization in media campaigns has little to do with actual concern about people’s health.  7. The average person gets little benefit from the popularization of health in media campaigns.  8. Health information in media campaigns has no impact on adult people’s behaviors.  9. Informing people about health in media campaigns is beneficial mostly to companies which offer health products and services.  10. Some health information in media campaigns encourages me to extend my knowledge.  11. When I encounter health information in media campaigns, it makes me curious.  12. I am glad when I encounter health information in media campaigns.  13. Health information is placed in media campaigns so that people know how to take care of their health.  14. Health information in media campaigns is beneficial to people who follow the recommendations.  15. Health information in media campaigns is necessary for all people. |
| Website Reputation | 1. This website that I seek health information has a good reputation.  2. This website that I seek health information has a good reputation compared to other rival websites.  3. This website that I seek health information has a reputation for offering good products or services.  4. This website that I seek health information has a reputation for being respectful to its customers. |
| Patient Adherence | 1. I am following/did follow the doctor’s suggestions exactly.  2. I am following/did follow the doctor’s drug/medication recommendations.  3. I am following/did follow the doctor's orders, such as to stay in bed.  4. I have returned or plan to return to the doctor on the schedule he/she suggested.  5. I have had or plan to have the follow-up tests recommended by the doctor |
